# Supplementary material for: Hydrogel oxygen reservoirs increase functional integration of neural stem cell grafts by meeting metabolic demands
Source: Nat Commun. 2023 Jan 28;14:457. doi: 10.1038/s41467-023-36133-8 (PMC9884236; doi:10.1038/s41467-023-36133-8)
Supplement: Supplementary file 1 — Supplementary Information [file 41467_2023_36133_MOESM1_ESM.pdf]

Supplementary Information

Hydrogel oxygen reservoirs increase functional integration of neural stem cell grafts by meeting metabolic demands

Y. Wang[1,2,3], E. R. Zoneff[1,3], J.W. Thomas[1], N. Hong[4], L. L. Tan[4], D. J. McGillivray[5], A. W. Perriman[6], K. C. L. Law[7], L. H. Thompson[7], N. Moriarty[7], C.L. Parish[7], R.J. Williams[8], C.J. Jackson[4,9,10], D.R. Nisbet[1,2,3]

[1]Laboratory of Advanced Biomaterials, Research School of Electrical, Energy and Materials Engineering, Australian National University, Canberra, ACT 2601, Australia

[2]The Graeme Clark Institute, The University of Melbourne, Parkville, VIC 3010, Australia

[3]Department of Biomedical Engineering, Faculty of Engineering and Information Technology, The University of Melbourne, Carlton, VIC 3053, Australia.

[4]Research School of Chemistry, Australian National University, Canberra, ACT 2601, Australia

[5]School of Chemical Sciences, University of Auckland, Private Bag 92019, Auckland 1142, New Zealand.

[6]School of Cellular and Molecular Medicine, University of Bristol, Bristol, Bristol BS8 1TD UK

[7]The Florey Institute of Neuroscience and Mental Health, The University of Melbourne, Parkville, VIC, Australia, 3052

[8]Institute for Mental and Physical Health and Clinical Translation, School of Medicine, Deakin University, Warun Ponds, VIC 3216, Australia

[9]Australian Research Council Centre of Excellence for Innovations in Peptide and Protein Science, Australian National University, Canberra, ACT 2601, Australia.

[10]Australian Research Council Centre of Excellence in Synthetic Biology, Australian National University, Canberra, ACT 2601, Australia.

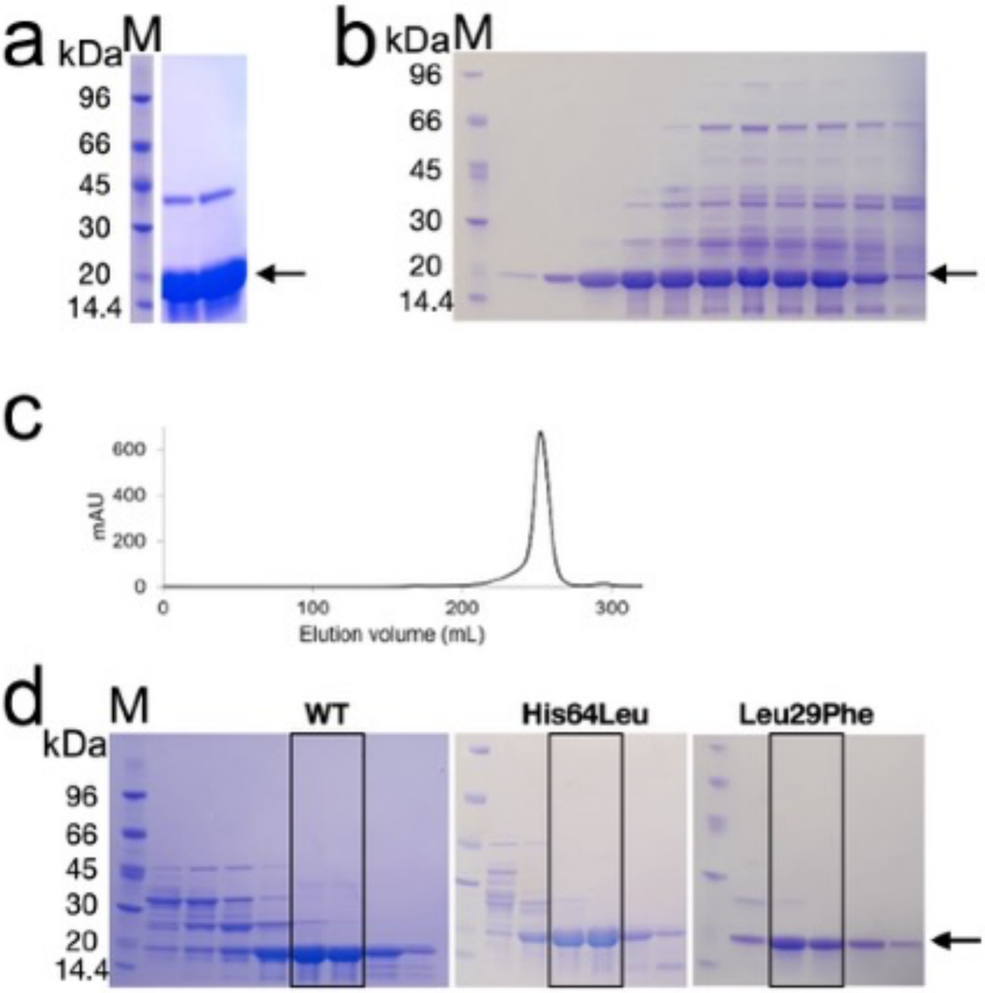

Supplementary Figure 1: Representative data for myoglobin purification. a SDS-PAGE of commercial *Equus caballus* myoglobin after size exclusion chromatography, a dominant band (>95%) at the correct molecular weight (~17 kDa) is visible as well as a minor band that corresponds to a small amount of dimer (~34 kDa). **b** SDS-PAGE of *Physeter macrocephalus* myoglobin after anion exchange chromatography. **c** Chromatogram of *Physeter macrocephalus* myoglobin from size exclusion. **d** SDS-PAGE of *Physeter macrocephalus* wild-type, His64Leu and Leu29Phe myoglobins after size exclusion chromatography. Pure fractions that were pooled for further use are boxed. The representative images in 1a,1b,1d show a summary of at least three independent experiments.

a

```
CLUSTAL W (1.83) multiple sequence alignment

MYG_PHYMC   MVLSEGEWQLVLHVWAKVEADVAGHGQDILIRLFKSHPETLEKFDRFKHLKTEAEMKASEDLKKHGVTVLTALGAILKKK
MYG_HORSE   MGLSDGEWQQVLNVWGKVEADIAGHGQEVLIRLFTGHPETLEKFDKFKHLKTEAEMKASEDLKKHGTVVLTALGGILKKK
            * **:**** **:**.*****:*****::*****..*********:*******************..******.*****

MYG_PHYMC   GHHEAELKPLAQSHATKHKIPIKYLEFISEAIIHVLHSRHPGDFGADAQGAMNKALELFRKDIAAKYKELGYQG
MYG_HORSE   GHHEAELKPLAQSHATKHKIPIKYLEFISDAIIHVLHSKHPGDFGADAQGAMTKALELFRNDIAAKYKELGFQG
            ****************************:********:*************.*******:**********:**
```

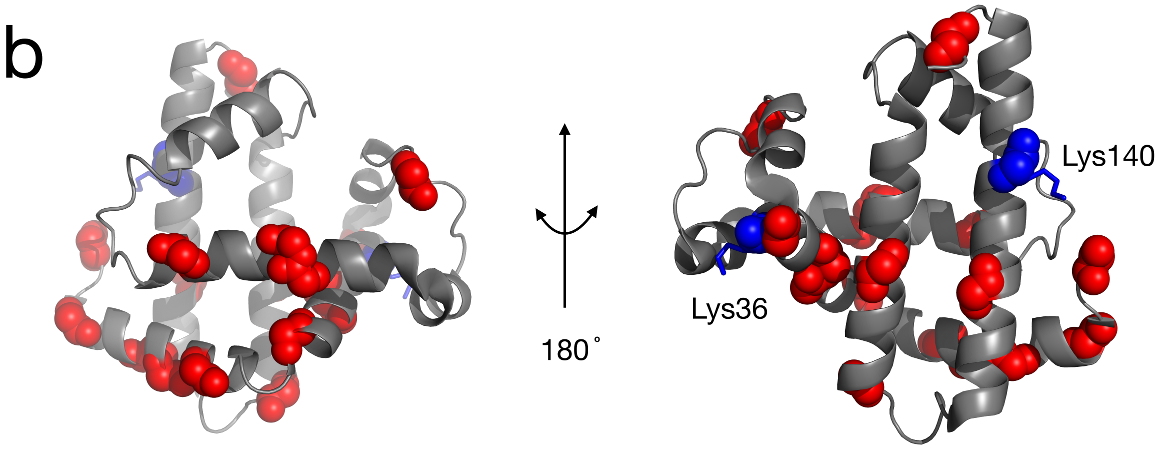

Supplementary Figure 2: a Sequence alignment between *Physeter macrocephalus* (sperm whale) myoglobin (MYGPHYMC) and *Equus caballus* (horse) myoglobin (MYGHORSE). There are only 12 amino acid differences between the sequences, most of which are conservative. There are two differences that make the MYGPHYMC more positively charged: Lys34Thr and Lys140Asn (blue), and 4 differences between polar and hydrophobic sidechains Leu10Gln, Ser35Gly, Val66Thr, Thr67Val, which are balanced between the sequences (2 polar>nonpolar *vs.* 2 nonpolar>polar). **b** Location of the sequence differences between *Physeter macrocephalus* and *Equus caballus* myoglobin. All mutations are surface exposed (red main chain atoms). The two sequence differences that contribute to higher positive surface charge (Lys36, Lys140) are shown in blue (main chain spheres and side chain sticks).

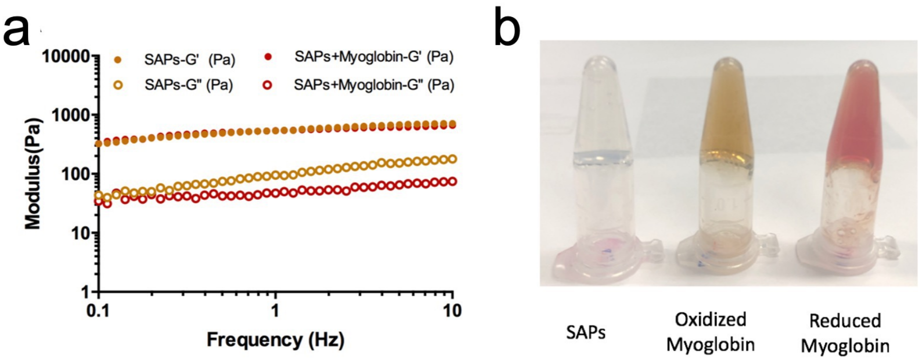

Supplementary Figure 3: Characterization of the SAP and SAP:myoglobin hydrogels. a Rheological analysis showing characteristic viscoelastic behavior; **b** Photograph of SAPs alone(left), oxidized myoglobin + SAPs (middle), and reduced myoglobin+SAPs(right: visible confirmation of reduction of myoglobin in SAPs by reaction with excess sodium dithionite. Source data are provided as a Source Data file.

$I(Q) \propto Q^{-\alpha}$	*α – Low-Q*	*α – Mid-Q*	*α – High-Q*
SAPs alone	2.30 ± 0.01	3.09 ± 0.01	1.40 ± 0.01
SAPs and myoglobin	2.03 ± 0.03	3.14 ± 0.01	1.62 ± 0.01

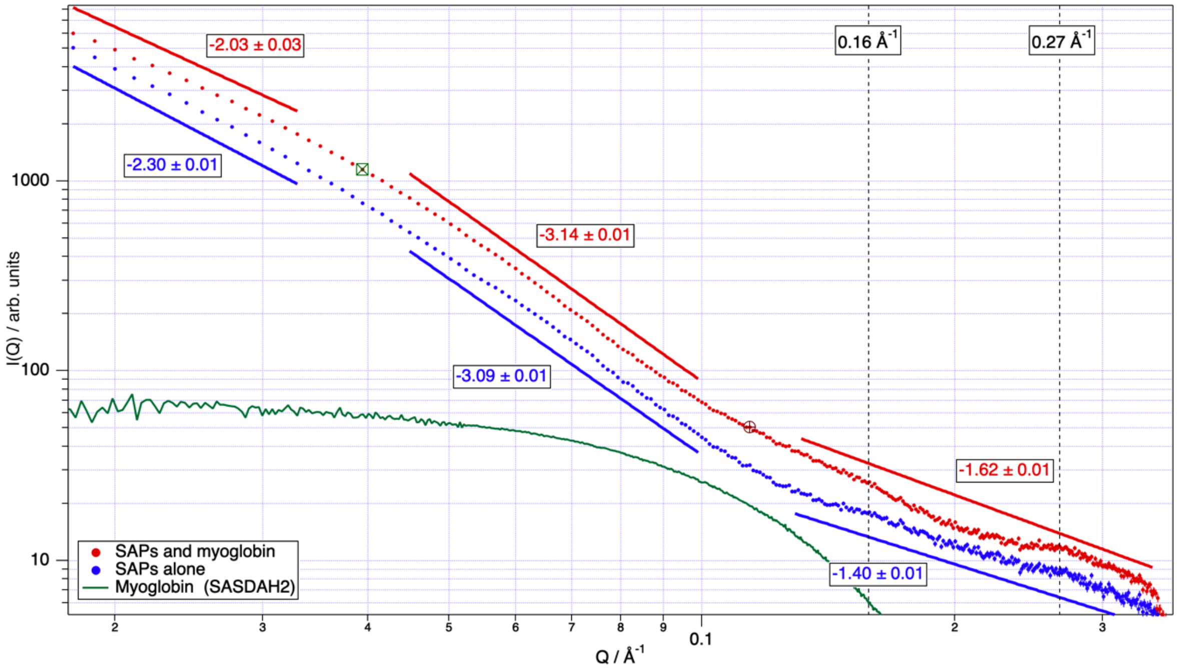

Supplementary Figure 4: SAP and SAP:Horse Myoglobin SAXS analysis. The SAP (blue) and SAP:horse myoglobin (red) samples are shown. Note that the two curves are offset for clarity. The scattering of myoglobin in solution (from the SAS database standard curve, https://www.sasbdb.org/data/SASDAH2/) is shown to indicate where its scattering would be significant. The scattering an low-, mid- and high-Q is shown, consistent with hydrogels and indicating random orientation of nanofibers in both samples and with minimal differences between the samples. Some weak scattering features at ~0.16 $Å^{-1}$ and 0.27 $Å^{-1}$ (marked with dashed lines; real space sizes 39 Å and 23 Å respectively) are present in both samples and likely reflect cluster sizes in the hydrogels, implying that the base structure of the SAPs is unaffected by inclusion of myoglobin. A slight difference between the two samples visible between 0.01 $Å^{-1}$ - 0.15 $Å^{-1}$ in the SAP:Mb sample, which is consistent with scattering from the myoglobin embedded in the SAPs network. Source data are provided as a Source Data file.

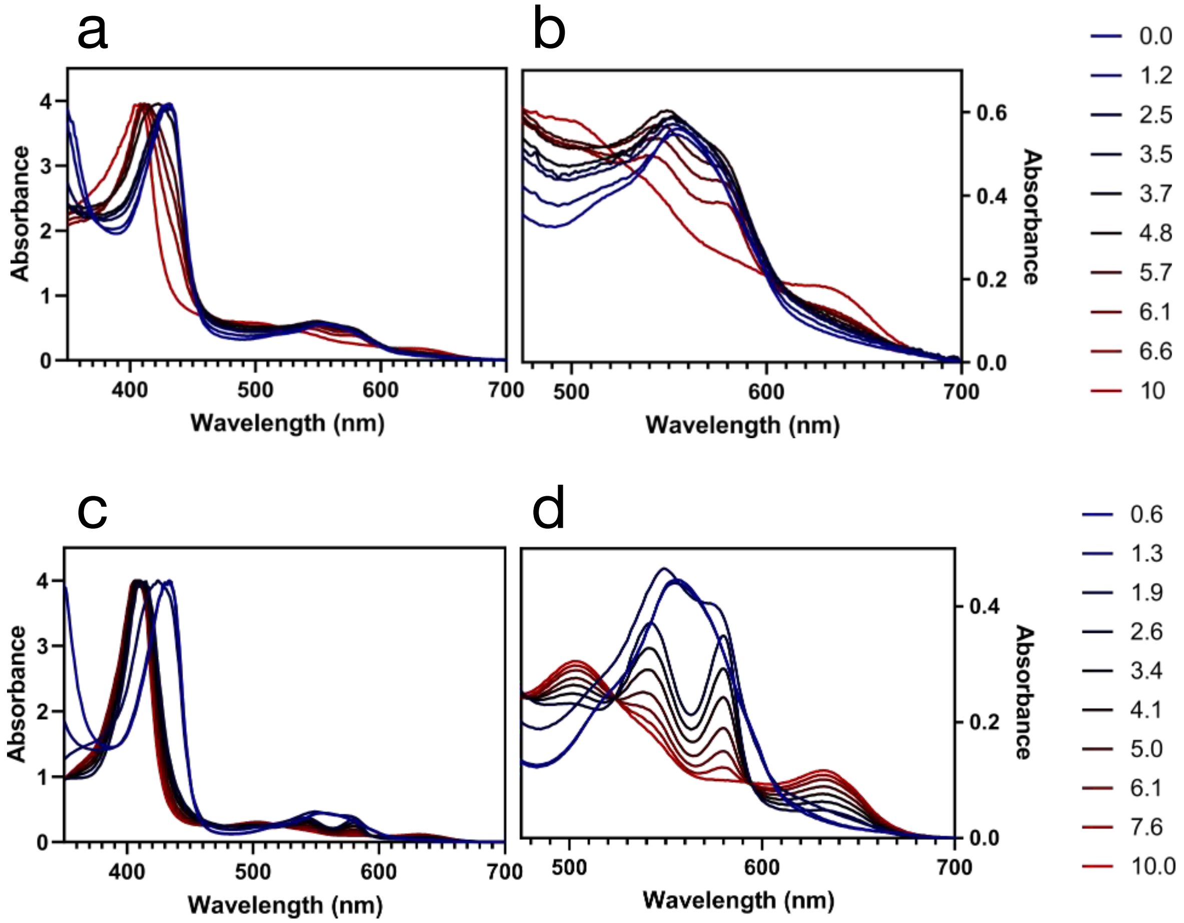

Supplementary Figure 5: Oxidation of horse myoglobin in the presence and absence of SAP hydrogel. a Functional analysis of the myoglobin:hydrogel over a 10 hour period, showing the gradual oxygenation and oxidation of myoglobin within the gel. Metmyoglobin (oxidized) is identifiable by a small peak at 628 nm, reduced absorbance between 550-600 nm, and a shift in the maximum absorbance from 426 to 409 nm. **b** Zoom on the region between 470-700 nm. **c** Functional analysis of reduced deoxymyoglobin in the absence of hydrogel over a 10 hour period, showing the formation of oxymyoglobin and oxidation of myoglobin to metmyoglobin (oxidized), which is identifiable by a small peak at 628 nm, reduced absorbance between 550-600 nm, and a shift in the maximum absorbance from 426 to 409 nm. The transition is significantly more rapid than for an the myoglobin:embedded in the SAP hydrogel. **d** Zoom on the region between 470-700 nm.

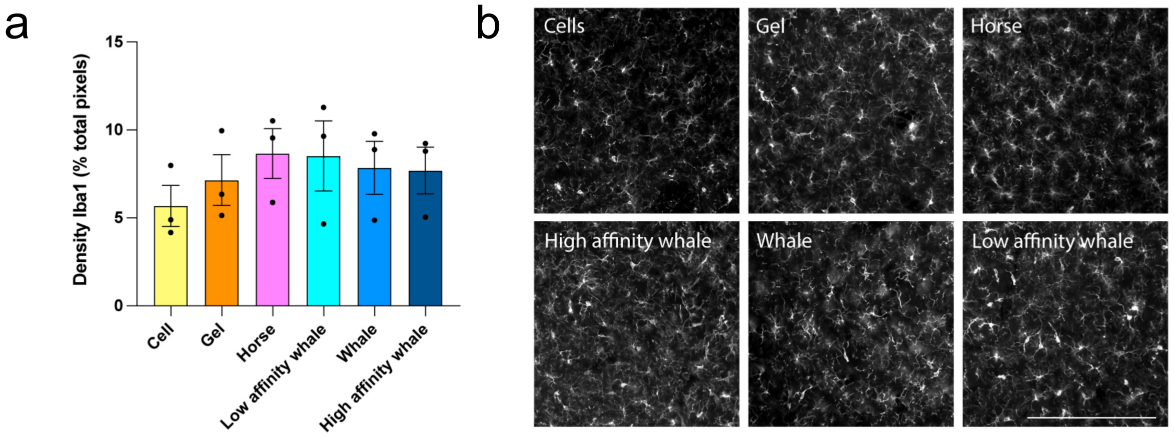

Supplementary Figure 6: Myoglobin had no effects on the host microglia response. a Density of Iba1+ microglia surrounding the GFP^{+} graft (n = 3). **b** Representative images of Iba1+ immunolabeling adjacent to GFP+ graft in SAPs and SAPs+Mbs groups, respectively. Data are presented as mean ± standard error of the mean (SEM). Scale bar represents 100 µm. The representative images in S6b show a summary of at least three independent experiments. Source data are provided as a Source Data file.

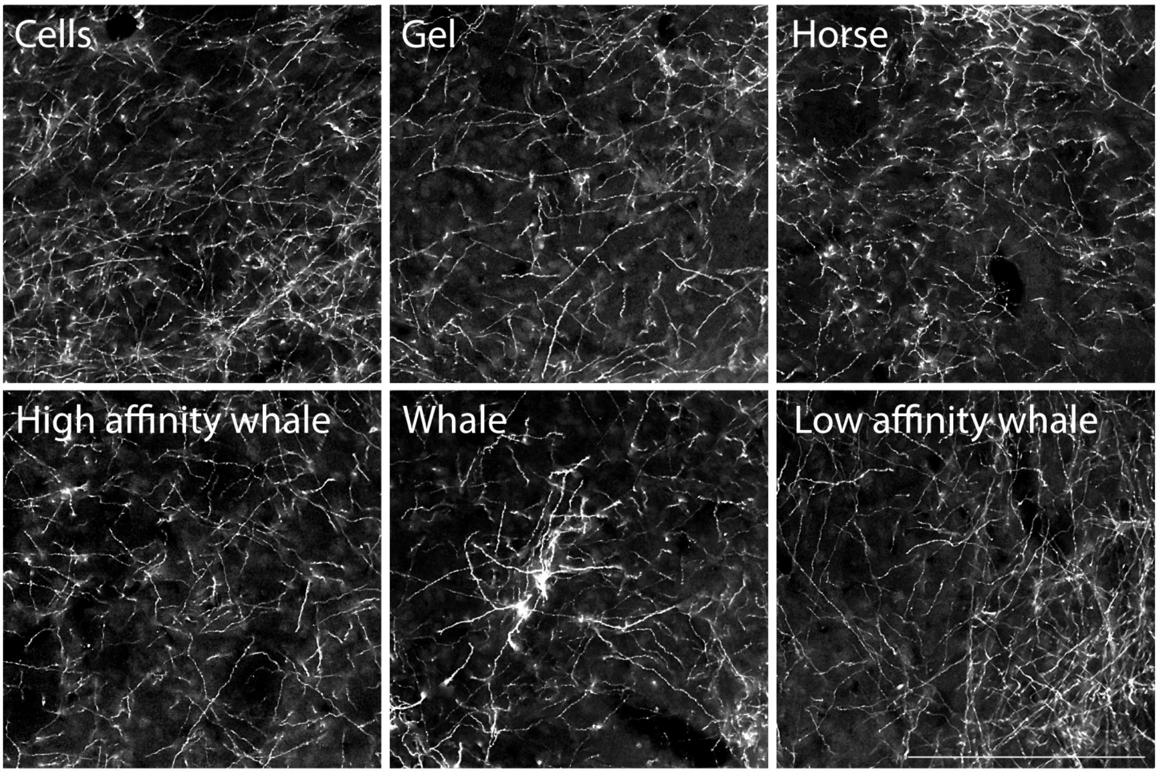

Supplementary Figure 7: Myoglobin-functionalised hydrogels support graft cell innervation and fibre extension. Representative images of GFP+ fibre density within host striatum tissue. Scale bar represents 100 µm. The representative images in S7 show a summary of at least three independent experiments.

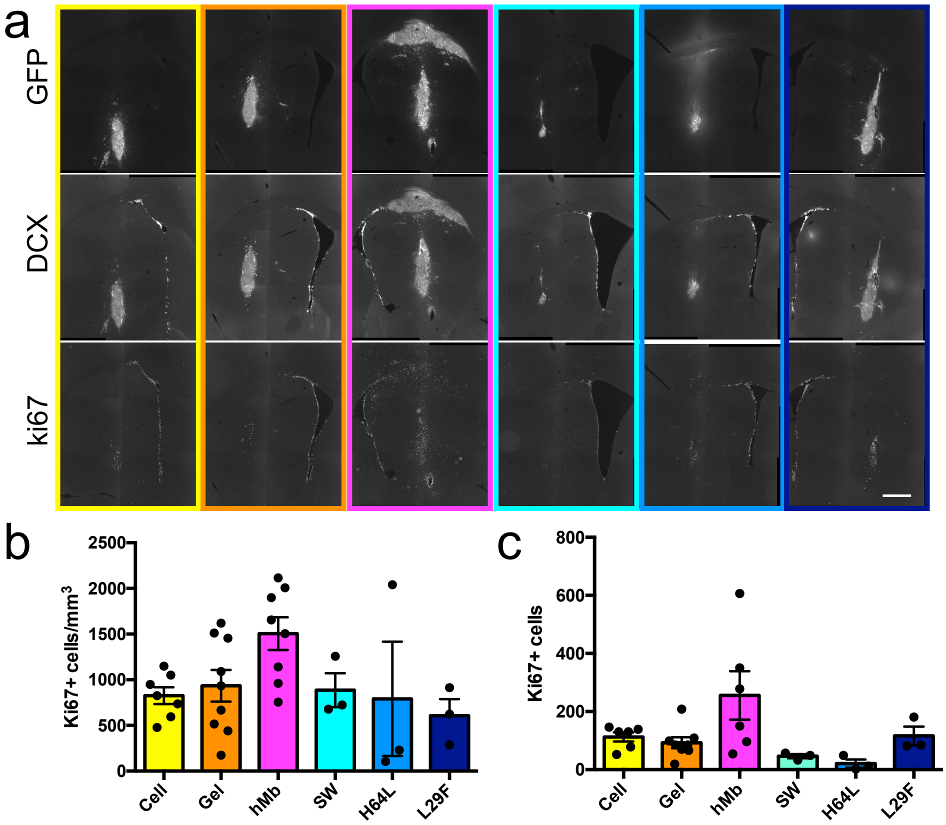

Supplementary Figure 8: Myoglobin has no effects on proliferation and migration. a Representative images of GFP+/ DCX+/Ki67 cells in in cell only, hydrogel and Mb:hydrogel groups, respectively. Scale bar represents 500 µm. The representative images in S8 show a summary of at least three independent experiments. **b** Density of Ki67+ proliferative cells in the graft in Cell (*n* = 7), Gel (*n* = 9), Horse (*n* = 8), Whale (*n*=3), Low affinity whale (*n* = 3) and High affinity whale (*n* = 3) groups. c Number of Ki67+ cells in graft in Cell (*n* = 6), Gel (*n* = 8), Horse (*n* = 6), Whale (*n* = 3), Low affinity whale (*n* = 3) and High affinity whale (*n* = 3) groups. Data are presented as mean ± standard error of the mean (SEM). Source data are provided as a Source Data file.

.
