# Supplementary material for: Hydrogel oxygen reservoirs increase functional integration of neural stem cell grafts by meeting metabolic demands
Source: Nat Commun. 2023 Jan 28;14:457. doi: 10.1038/s41467-023-36133-8 (PMC9884236; doi:10.1038/s41467-023-36133-8)
Supplement: Supplementary file 3 — Reporting Summary [file 41467_2023_36133_MOESM3_ESM.pdf]

nature portfolio

Corresponding author(s): David Nisbet
Last updated by author(s): Jan 12, 2023

Reporting Summary

Nature Portfolio wishes to improve the reproducibility of the work that we publish. This form provides structure for consistency and transparency in reporting. For further information on Nature Portfolio policies, see our Editorial Policies and the Editorial Policy Checklist.

Statistics

For all statistical analyses, confirm that the following items are present in the figure legend, table legend, main text, or Methods section.

n/a	Confirmed	
☐	☒	The exact sample size (*n*) for each experimental group/condition, given as a discrete number and unit of measurement
☐	☒	A statement on whether measurements were taken from distinct samples or whether the same sample was measured repeatedly
☐	☒	The statistical test(s) used AND whether they are one- or two-sided<br>*Only common tests should be described solely by name; describe more complex techniques in the Methods section.*
☐	☒	A description of all covariates tested
☐	☒	A description of any assumptions or corrections, such as tests of normality and adjustment for multiple comparisons
☐	☒	A full description of the statistical parameters including central tendency (e.g. means) or other basic estimates (e.g. regression coefficient) AND variation (e.g. standard deviation) or associated estimates of uncertainty (e.g. confidence intervals)
☐	☒	For null hypothesis testing, the test statistic (e.g. *F*, *t*, *r*) with confidence intervals, effect sizes, degrees of freedom and *P* value noted<br>*Give P values as exact values whenever suitable.*
☒	☐	For Bayesian analysis, information on the choice of priors and Markov chain Monte Carlo settings
☒	☐	For hierarchical and complex designs, identification of the appropriate level for tests and full reporting of outcomes
☒	☐	Estimates of effect sizes (e.g. Cohen's *d*, Pearson's *r*), indicating how they were calculated

Our web collection on statistics for biologists contains articles on many of the points above.

Software and code

Policy information about availability of computer code

Data collection	The sequences of Equus caballus (Horse Mb,S wissProt accession number: P68 082) and Physeter macrocephalus (Sperm whale Mb,S wissProt Data analysis accession number: P02185) were aligned with T-COFFEE server and the pI values were calculated using the Expasy ProtParam Tool.Chirascan software (Version 4.5.1848.0) was used to average and smooth Circular dichroism data obtained using a Chirascan CD Spectrometer(Applied Photophisics Limited,version4.5.1848.0). ScatterBrain was used to average and subtract the PBS background from SAXS data. rSpace (Malvern, version 1.72) was used to perform the rheological analysis collected from a Kinexus Pro+ Rheometer (Malvern).
Data analysis	Data were analyzed using Graph Pad Prism 6 .0 by one-way ANOVA with Turkey post-hoc statistic testing for multiple comparisons or by a two tailed t-test. For analysis of lbal + labelled cells,a one-way ANOVA with post hoc Bonferroni was used.

For manuscripts utilizing custom algorithms or software that are central to the research but not yet described in published literature, software must be made available to editors and reviewers. We strongly encourage code deposition in a community repository (e.g. GitHub). See the Nature Portfolio guidelines for submitting code & software for further information.

Data

Policy information about availability of data

All manuscripts must include a data availability statement. This statement should provide the following information, where applicable:

- Accession codes, unique identifiers, or web links for publicly available datasets
- A description of any restrictions on data availability
- For clinical datasets or third party data, please ensure that the statement adheres to our policy

The atomic coordinates and structure factors for the Leu29Phe mutant of Physeter macrocephalus myoglobin (High affinity whale Mb, PDB ID: 2SPL), wild-type Physeter macrocephalus myoglobin (Sperm whale Mb, PDB ID: 1VXC),the His64Leu mutant of Physeter macrocephalus myoglobin (Low affinity whale Mb, PDB ID: 2MGE) are available from the Worldwide Protein Data Bank (http://wwpdb.org/). All data during and/or analysed during the current study are available within the article and Supplementary Files, or available from the corresponding authors on reasonable request. The source data underlying all Figures and Supplementary Figures are provided as a Source Data file.

Human research participants

Policy information about studies involving human research participants and Sex and Gender in Research.

Reporting on sex and gender	Information on sex and gender has not been collected.
Population characteristics	Information on population characteristics has not been collected.
Recruitment	No human research participants were recruited.
Ethics oversight	Ethics approval for human research was not required.

Note that full information on the approval of the study protocol must also be provided in the manuscript.

Field-specific reporting

Please select the one below that is the best fit for your research. If you are not sure, read the appropriate sections before making your selection.

☒ Life sciences ☐ Behavioural & social sciences ☐ Ecological, evolutionary & environmental sciences

For a reference copy of the document with all sections, see nature.com/documents/nr-reporting-summary-flat.pdf

Life sciences study design

All studies must disclose on these points even when the disclosure is negative.

Sample size	To mimimise the death of animals the sample size was kept small (n= 6). This allowed one sample for each variable and control with appropriate significance with the ANOVA testing performed.
Data exclusions	Animals that had their graft outside of the striatum (i.e. mis-targeted graft placement) were excluded from analysis.
Replication	Not applicable.
Randomization	Animals were randomly allocated into experimental groups and images were taken at random locations within brains slices before statistical analysis was performed on the images.
Blinding	At the time of data collection (inclusive of graft volumetric assessments and cell quantifications), researchers were blinded to the experimental conditions.

Reporting for specific materials, systems and methods

We require information from authors about some types of materials, experimental systems and methods used in many studies. Here, indicate whether each material, system or method listed is relevant to your study. If you are not sure if a list item applies to your research, read the appropriate section before selecting a response.

Materials & experimental systems

n/a	Involved in the study
☐	☒ Antibodies
☐	☒ Eukaryotic cell lines
☒	☐ Palaeontology and archaeology
☐	☒ Animals and other organisms
☒	☐ Clinical data
☒	☐ Dual use research of concern

Methods

n/a	Involved in the study
☒	☐ ChIP-seq
☒	☐ Flow cytometry
☒	☐ MRI-based neuroimaging

Antibodies

Antibodies used

rabbit anti-GFP (1: 20,000; Abcam, ab290), chicken anti-GFP (1: 1,000; Abcam, ab13970), sheep anti-Ki67 (1:40, R&D Systems, AF7649), goat anti-doublecortin(DCX) (1:1000, Santa Cruz, sc-8066), chicken anti-GFAP (anti-glial fibrillary acidic protein, 1:500, Novus biological, NBP1-056198), mouse anti-NeuN (1:1000;Abcam, ab104224), rabbit anti-iba1 (1:1000, WAKO, 019-19741) and biotin conjugated donkey anti-rabbit (1:500; Jackson ImmunoResearch, 711-065-152).

Validation

rabbit anti-GFP (1: 20,000; Abcam, ab290): host species- rabbit, reacts with: species independent; suitable for ELISA, IHC-Fr, ICC, IHC-P, IP, WB, IHC-FoFr, IHC-FrFl, Electron Microscopy (https://www.abcam.com/gfp-antibody-ab290.html)
chicken anti-GFP (1: 1,000; Abcam, ab13970):host species-chicken, reacts with: species independent, suitable for WB, ICC/IF (https://www.abcam.com/gfp-antibody-ab13970.html)
sheep anti-Ki67 (1:40, R&D Systems, AF7649):host species-sheep, species reactivity-mouse; suitable for ICC, IHC, IHC-P (https://www.rndsystems.com/products/mouse-ki67-mki67-antibody_af7649#product-details)
goat anti-doublecortin (DCX) (1:1000, Santa Cruz, sc-8066): host species-goat, species reactivity-mouse; suitable for WB, immunoprecipitation, and immune fluorescence (IHC) (https://www.scbt.com/p/doublecortin-antibody-c-18)
chicken anti-GFAP (anti-glial fibrillary acidic protein, 1:500, Novus biological, NBP1-056198): host species-chicken, species reactivity-mouse, rat, human; suitable for ICC/IF, IHC, IHC-F, IHC-Fr,MI, WB (https://www.novusbio.com/products/gfap-antibody_nbp1-05198#reviews-publications)
mouse anti-NeuN (1:1000;Abcam, ab104224): host species-mouse, species reactivity-mouse, rat, human; suitable for ICC, IHC-P, WB (https://www.abcam.com/neun-antibody-1b7-neuronal-marker-ab104224.html)
rabbit anti-iba1 (1:1000, WAKO, 019-19741): host species-rabbit, species reactivity-mouse, rat, human; suitable for ICC, IHC(Frozen) (https://labchem-wako.fujifilm.com/asia/product/detail/W01W0101-1974.html)
biotin conjugated donkey anti-rabbit (1:500; Jackson ImmunoResearch, 711-065-152): host species-donkey, species reactivity-rabbit; suitable for flow cytometry and fluorescence immunohisto/cytochemistry, enzyme immunohisto/cytochemistry, ELISA and Western blotting using enzyme-conjugated streptavidin
(https://www.jacksonimmuno.com/catalog/products/711-065-152)

Eukaryotic cell lines

Policy information about cell lines and Sex and Gender in Research

Cell line source(s)

Cell for grafting were obtained from primary cortical tissue, isolated from embryonic day 14.5 (E14.5) embryos (both male and female). Embryos were generated from time-mated mice with green fluorescent protein (GFP) expressed under the β-actin promoter. Mycoplasma was not present in the colonies.

Authentication

The cells were obtained from primary dissection.

Mycoplasma contamination

Primary cells were used.

Commonly misidentified lines
(See ICLAC register)

No commonly misidentified lines were used.

Animals and other research organisms

Policy information about studies involving animals; ARRIVE guidelines recommended for reporting animal research, and Sex and Gender in Research

Laboratory animals

Animals were group housed in individually ventilated cages with ad libtum access to food and water. Cotical brain tissue was isolated from mice pups at embryonic day14.5. Implantations were made to adult C57BL/6 mice (n=6).

Wild animals

The study did not involve wild animals.

Reporting on sex

Sex was not considered in the study design.

Field-collected samples

The study does not involve samples collected from the field.

Ethics oversight

All animal procedures and methods were conducted in accordance with the Australian National Health and Medical Research Council's published Code of Practice for the Use of Animals in Research and were approved by the Florey Institute of Neuroscience

and Mental Health Animal Ethics Committee (protocol code 18-005, February 2018).

Note that full information on the approval of the study protocol must also be provided in the manuscript.
